# Polymyalgia Rheumatica (PMR) and Polymyalgia Rheumatica-like (PMR-like) Manifestations in Cancer Patients Following Treatment with Nivolumab and Pembrolizumab: Methodological Blurred Points Identified Through a Systematic Review of Published Case Reports

**DOI:** 10.3390/medsci13020034

**Published:** 2025-04-01

**Authors:** Ciro Manzo, Marco Isetta, Alberto Castagna, Melek Kechida

**Affiliations:** 1 Rheumatologic Outpatient Clinic, Department of Internal and Geriatric Medicine, Azienda Sanitaria Locale Napoli 3, 80065 Sant’Agnello, Italy; 2 Library and Knowledge Services, Central and North West London NHS Foundation Trust, London UB8 3NN, UK; marco.isetta@nhs.net; 3 Department of Primary Care, Health District of Soverato, Azienda Sanitaria Provinciale Catanzaro, 88100 Catanzaro, Italy; albertocastagna78@gmail.com; 4 Department of Internal Medicine and Endocrinology, Fattouma Bourguiba University Hospital, Faculty of Medicine, University of Monastir, Monastir 5000, Tunisia; kechida_mel_lek@hotmail.com

**Keywords:** polymyalgia rheumatica, immunotherapy, immune checkpoints inhibitors, systematic review, adverse drug reaction, case reports, nivolumab, pembrolizumab, Naranjo’s scale, methodology

## Abstract

**Background**: Among rheumatologic diseases following therapy with immune checkpoint inhibitors (ICIs), the cases of cancer patients diagnosed as having polymyalgia rheumatica (PMR), particularly with nivolumab and pembrolizumab, has been steadily rising in published reports. **Objectives**: We performed a systematic review of published case reports with the aim of answering these questions: (1) Is PMR following therapy with nivolumab and pembrolizumab an adverse drug reaction (ADR)? (2) Is there a difference between cases of PMR following therapy with nivolumab and those following therapy with pembrolizumab? **Methods**: Based on Preferred Reporting Items for Systematic Reviews and Meta-Analysis (PRISMA) guidelines, a comprehensive literature search in three main bibliographic databases: MEDLINE (Ovid interface), EMBASE, and COCHRANE Library was carried out on 27 December 2024. This systematic review has no registration number. Results: Data were extracted from 12 patients. Namely, 5 cases followed treatment with nivolumab and 7 with pembrolizumab. Validated scales for ADR assessment—such as Naranjo’s scale—were not used in 10 out of the 12 patients. Additionally, validated diagnostic or classification criteria for PMR were used in the majority of case reports related to nivolumab. On the contrary, clinical judgment alone was the rule in almost all case reports on pembrolizumab. Finally, the time interval between PMR manifestations and nivolumab/pembrolizumab therapy ranged from one to 14 cycles (fully compatible with pharmacokinetics). **Conclusions**: Our literature review highlighted significant methodological blurred lines in the categorization of PMR following therapy with nivolumab or pembrolizumab.

## 1. Background

Polymyalgia rheumatica (PMR) is one of the most common inflammatory rheumatic diseases affecting elderly patients [1,2,3,4]. Typically, patients with PMR complain of sudden and disabling pain in both the shoulders and pelvic girdle, associated with long-lasting morning stiffness (>45 min). Low-grade fever, loss of appetite and weight, and sleeping disorders can be additionally present in some patients. Erythrocyte sedimentation rate (ESR) and C-reactive protein (CRP) serum concentrations) are usually raised at the time of diagnosis [5,6,7]. However, a proper diagnosis of PMR is also possible even if ESR (values disaggregated by gender and age [8]) and CRP are in their normal range, provided that typical manifestations are present and mimicking conditions are excluded [9,10,11,12,13,14]. Additionally, shoulder and hip ultrasound (US) findings have been proposed to improve the classification specificity [15]. Finally, a fast and significant improvement following <15–20 mg/day prednisone with the reappearance of symptoms when prednisone is stopped too early is commonly used in everyday clinical practice as an ex adiuvantibus criterion [16,17,18].

Similar symptoms and clinical signs—as well as a favorable response to glucocorticoids—may, however, be present in several other different conditions or diseases, so drawing a boundary line between PMR and all the conditions that may be confused and exchanged for PMR, including cancer and paraneoplastic syndromes, is anything but straightforward [19,20,21,22,23,24].

In 2011, the Food and Drug Administration (FDA) approved the treatment with immune checkpoint inhibitors (ICIs) in patients with metastatic melanoma. To date, therapy with ICIs can be proposed in progressively increasing types of cancers [25,26,27,28,29,30]. The number of cases of cancer patients developing PMR, particularly with nivolumab and pembrolizumab, has been increasing [31,32]. However, different study types can affect conclusions that are often disjointed and sometimes misleading. For example, studies from institutional databases tend to rely on coding-based diagnoses of diseases, which are not routinely confirmed by the reviews of individual medical records. Consequently, these studies could easily result in misdiagnosis/misclassification. Case reports, on the other hand, better capture what happens to the individual patient because all data is detailed, so providing feedback on clinical practice guidelines and offering a framework for early signals of adverse events [33,34,35].

Nivolumab and pembrolizumab (approved in 2015 and in 2017, respectively) are fully human IgG4 antibodies directed against the PD-1 receptor. PD-1 is a regulatory receptor located on the lymphocyte surface. It is involved in maintaining the balance between T lymphocyte activation and inhibition/suppression, preventing—as checkpoints—the immune system from attacking itself. Specifically, nivolumab and pembrolizumab binding to the PD-1 receptor blocks any possible interaction between the PD-1 receptor and its ligand PD-L1. Consequently, inactivation of T-cells—mainly CD8+ T lymphocytes—does not occur. As a result, lymphocyte action against antigens present in the cancerous cells is enhanced, and this contributes to their destruction [36,37,38]. Unfortunately, this same interaction can trigger adverse inflammatory (both autoinflammatory and immune-mediated) events.

In this context, a systematic review of published case reports was conducted with the aim of trying to answer these two questions: (1) Are PMR or PMR-like syndromes following therapy with nivolumab or pembrolizumab an adverse drug reaction (ADR)? (2) Is there a difference between cases of PMR following therapy with nivolumab and those following therapy with pembrolizumab?

## 2. Materials and Methods

Our systematic review was based on Preferred Reporting Items for Systematic Reviews and Meta-Analysis (PRISMA) guidelines [39]. There was no registration number.

### 2.1. Search Strategy

One of the authors (Isetta, M) performed a state-of-the-art bibliographic search in three main bibliographic databases: MEDLINE (Ovid interface), EMBASE, and COCHRANE Library, using the following main search terms: polymyalgia rheumatica, immune checkpoint blockade, rheumatic syndromes, checkpoint inhibitors therapy, polymyalgia rheumatica-like syndromes, immunotherapy, checkpoint inhibitor-associated polymyalgia rheumatica, anti-PD-1, anti-PD-1 antibody, and anti-programmed death 1 monoclonal antibody (both MESH headings and free text). Searches were carried out on 27 December 2024. Each paper’s reference list was scanned for additional publications meeting this study’s aim. When papers reported data partially presented in previous articles, we referred to the most recent published data. After de-duplication, all retrieved studies were examined. In accordance with the PRISMA 2009 checklist, the full search strategy for one database (Ovid MEDLINE) is detailed in Supplementary Materials (File S1).

### 2.2. Inclusion Criteria

We included all case reports published after 2015 (when the FDA approved the use of nivolumab) describing PMR and PMR-like manifestations following treatment with nivolumab and pembrolizumab.

### 2.3. Exclusion Criteria

Conference abstracts, comments, and secondary articles were excluded. The presence of giant cell arteritis (GCA) was an additional exclusion criterion unless all data concerning GCA were clearly distinct from PMR findings. Indeed, GCA (a large-vessel vasculitis potentially associated with PMR) may have a neoplastic risk in itself, especially for specific types of cancers [40]. Therefore, assessing these patients together may lead to methodological bias, notably with regard to a more frequent association with cancer.

Other exclusion criteria were as follows:

(a) Non-English language reports when English abstract was lacking;
(b) case reports on nivolumab or pembrolizumab used in association with other ICIs (so-called “combo-therapy”) and when used in unapproved conditions;
(c) Case reports were assessed as being of poor quality according to the 2007 International Society for Pharmacoepidemiology (ISPE) and the International Society of Pharmacovigilance (ISoP) guidelines [41].

### 2.4. Data Extraction

All article titles identified were screened by one author (Isetta, M) against the inclusion and exclusion criteria. Subsequently, two other authors (Manzo, C and Castagna, A) independently reviewed the titles and abstracts of all identified records. When case reports were written in a non-English language, the English abstract was considered. Full-text versions of potentially relevant papers were then sourced. Finally, reasons for exclusion were recorded, and disagreements were resolved by consensus among all authors.

The same reviewers used a standardized ad hoc form (Supplementary Material, File S2) to independently extract all pertinent data, such as diagnostic or classification criteria adopted to diagnose PMR (if and when specified), the time interval between PMR and nivolumab or pembrolizumab if and how PMR and PMR-like conditions were evaluated as an ADR. Meta-data from each case report, such as lead author name and publication year, were also listed.

### 2.5. Quality Assessment

The methodological quality of the included reports was independently assessed by all the authors using the guidelines for submitting adverse reports for publication endorsed in 2007 by ISPE and ISoP. In view of one of the objectives of this review (that is to assess if PMR following therapy with nivolumab or pembrolizumab can always be considered as an ADR), particular attention was given to the ISPE/ISoP Drug and to the Adverse Event categories: identification, dosage, administration, concomitant therapies (if any), and ADR description. Specifically, ADR identification and description took account of the scale proposed by Naranjo et al. for estimating whether an ADR is actually due to the drug or other factors (the disease itself, for example). According to this scale, four levels of ADR probability are possible: definite ADR if the total score is ≥9; probable ADR if the total score is between 5 and 8; possible ADR if the total score is between 1 and 4; doubtful ADR if the total score is 0. The higher the score (the range is from −4 to +13), the higher the probability of an ADR [42].

With particular regard to this specific assessment, all data present in case reports were carefully examined without any restrictions. Disagreements were discussed and settled by consensus.

## 3. Results

### 3.1. Description of Included Studies

**Figure 1:**
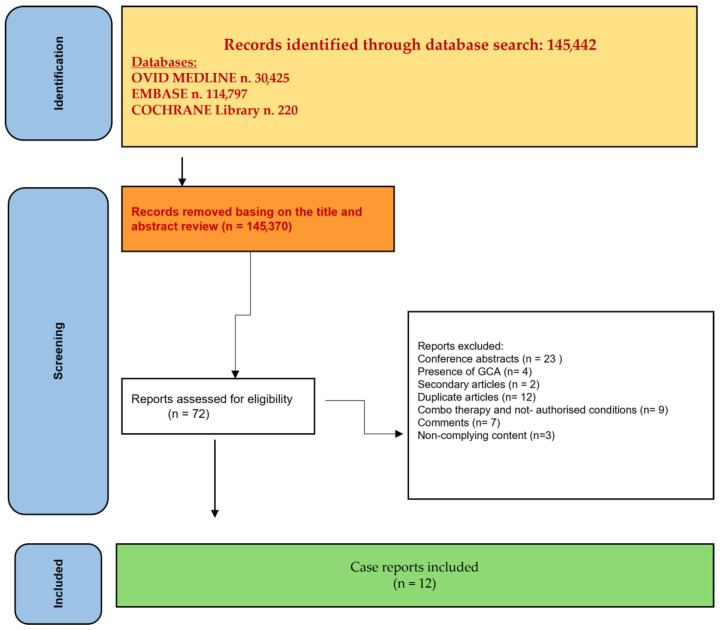
PRISMA flowchart of the included case reports.

An overview of the study identification process is reported in Figure 1. The initial search yielded 145,442 papers, of which 145,370 articles were excluded based on title and abstract screening. A total of 72 articles were then assessed for eligibility and underwent a full-length review. Finally, 12 case reports were included.

### 3.2. Case Reports

Table 1 and Table 2 reported the main characteristics of the included case reports in accordance with the specific objectives of this systematic review.

Naranjo’s scale values were estimated on the basis of the information made available by the authors, as follows: BERNIER: (1) There are previous conclusive reports on this reaction (+1); (2) The adverse event appeared after the suspected drug was given (+2); (3) The adverse reaction improved when the drug was discontinued and a specific antagonist was given (+1); (4) There was no drug reintroduction (0); (5) Exclusion of alternative causes that could have caused the reaction (+2); (6) A placebo was not given (0); (7) the drug was not detected in any body fluid in toxic concentrations (0); (8) No dosage modification (0); (9) The patient did not have a similar reaction to the same drug in previous exposure (0); (10)The adverse event was confirmed by objective evidence (+1). NAKAMAGOE: (1) There are previous conclusive reports on this reaction (+1); (2) The adverse event appeared after the suspected drug was given (+2); (3) The adverse reaction improved when the drug was discontinued, and a specific antagonist was given (+1); (4) There was no drug reintroduction (0); (5) Exclusion of alternative causes that could have caused the reaction (+2); (6) A placebo was not given (0); (7) the drug was not detected in any body fluid in toxic concentrations (0); (8) No dosage modification (0); (9) The patient did not have a similar reaction to the same drug in previous exposure (0); (10)The adverse event was confirmed by objective evidence (+1). IMAY: (1) There are previous conclusive reports on this reaction (+1); (2) The adverse event appeared after the suspected drug was given (+2); (3) The adverse reaction improved when the drug was discontinued, and a specific antagonist was given (+1); (4) There was no drug reintroduction (0); (5) Exclusion of alternative causes that could have caused the reaction (+2); (6) A placebo was not given (0); (7) the drug was not detected in any body fluid in toxic concentrations (0); (8) No dosage modification (0); (9) The patient did not have a similar reaction to the same drug in previous exposure (0); (10)The adverse event was confirmed by objective evidence (+1). LOBO: (1) There are previous conclusive reports on this reaction (+1); (2) The adverse event did not appear after the suspected drug was given (−1); (3) The adverse reaction improved when a specific antagonist was given (+1); (4) The drug was not re-administered (0); (5) There was alternative causes (idiopathic PMR) that could have caused the manifestations (−1); (6) A placebo was not given (0); (7) the drug was not detected in any body fluid in toxic concentrations (0); (8) No dosage modification (0); (9) The patient did not have a similar reaction to the same drug in previous exposure (0); (10)The adverse event was not confirmed by objective evidence (0). MOURA: (1) There are previous conclusive reports on this reaction (+1); (2) The adverse event appeared after the suspected drug was given (+2); (3) The adverse reaction improved when the drug was discontinued and a specific antagonist was given (+1); (4) Drug reintroduction was not clear (0); (5) No exclusion of alternative causes that could have caused the reaction (0); (6) A placebo was not given (0); (7) the drug was not detected in any body fluid in toxic concentrations (0); (8) No dosage modification (0); (9) The patient did not have a similar reaction to the same drug in previous exposure (0); (10)The adverse event was not confirmed by objective evidence (0).

Naranjo’s scale estimated value was based on data available in the listed case reports, as follows: GAREL-1: (1) There are previous conclusive reports on this reaction (+1); (2) The adverse event appeared after the suspected drug was given (+2); (3) The adverse reaction improved when the drug was discontinued and a specific antagonist was given (+1); (4) Drug reintroduction was not clear (0); (5) Exclusion of alternative causes that could have caused the reaction was not reported (0); (6) A placebo was not given (0); (7) the drug was not detected in any body fluid in toxic concentrations (0); (8) Dosage modification was not clear (0); (9) The patient did not have a similar reaction to the same drug in previous exposure (0); (10)The adverse event was confirmed by objective evidence (+1). GAREL-2: (1) There are previous conclusive reports on this reaction (+1); (2) The adverse event appeared after the suspected drug was given (+2); (3) The adverse reaction improved when the drug was discontinued, and a specific antagonist was given (+1); (4) Drug reintroduction was not reported (0); (5) Exclusion of alternative causes that could have caused the reaction was not reported (0); (6) A placebo was not given (0); (7) the drug was not detected in any body fluid in toxic concentrations (0); (8)Dosage modification was not clear (0); (9) The patient did not have a similar reaction to the same drug in previous exposure (0); (10)The adverse event was confirmed by objective evidence (+1). KUSWANTO: (1) There are previous conclusive reports on this reaction (+1); (2) The adverse event appeared after the suspected drug was given (+2); (3) The adverse reaction improved when the drug was discontinued and a specific antagonist was given (+1); (4) Drug reintroduction was not reported (0); (5) Exclusion of alternative causes that could have caused the reaction was not reported (0); (6) A placebo was not given (0); (7) the drug was not detected in any body fluid in toxic concentrations (0); (8) Dosage modification (+1); (9) The patient did not have a similar reaction to the same drug in previous exposure (0); (10)The adverse event was confirmed by objective evidence (+1). ISKANDAR: (1) There are previous conclusive reports on this reaction (+1); (2) The adverse event appeared after the suspected drug was given (+2); (3) The adverse reaction improved when the drug was discontinued and a specific antagonist was given (+1); (4) Drug reintroduction was not reported (0); (5) Exclusion of alternative causes that could have caused the reaction was not reported (0); (6) A placebo was not given (0); (7) the drug was not detected in any body fluid in toxic concentrations (0); (8) Dosage modification (+1); (9) The patient did not have a similar reaction to the same drug in previous exposure (0); (10)The adverse event was confirmed by objective evidence (+1).  ROBILLIARD: (1) There are previous conclusive reports on this reaction (+1); (2) The adverse event appeared after the suspected drug was given (+2); (3) The adverse reaction improved when the drug was discontinued and a specific antagonist was given (+1); (4) Drug reintroduction was not reported (0); (5) Exclusion of alternative causes that could have caused the reaction was not reported (0); (6) A placebo was not given (0); (7) the drug was not detected in any body fluid in toxic concentrations (0); (8) Dosage modification (+1); (9) The patient did not have a similar reaction to the same drug in previous exposure (0); (10)The adverse event was confirmed by objective evidence (+1). PERROTTA: (1) There are previous conclusive reports on this reaction (+1); (2) The adverse event appeared after the suspected drug was given (0); (3) The adverse reaction improved when the drug was discontinued, and a specific antagonist was given (+1); (4) Drug reintroduction was not reported (0); (5) Exclusion of alternative causes that could have caused the reaction was not reported (0); (6) A placebo was not given (0); (7) the drug was not detected in any body fluid in toxic concentrations (0); (8) Dosage modification (+1); (9) The patient did not have a similar reaction to the same drug in previous exposure (0); (10)The adverse event was confirmed by objective evidence (+1). KETHIREDDY: (1) There are previous conclusive reports on this reaction (+1); (2) The adverse event appeared after the suspected drug was given (+2); (3) The adverse reaction improved when the drug was discontinued, and a specific antagonist was given (+1); (4) Drug reintroduction was not reported (0); (5) Exclusion of alternative causes that could have caused the reaction was not reported (0); (6) A placebo was not given (0); (7) the drug was not detected in any body fluid in toxic concentrations (0); (8) Dosage modification (+1); (9) The patient did not have a similar reaction to the same drug in previous exposure (0); (10)The adverse event was confirmed by objective evidence (+1).

## 4. Discussion

Based on our literature search strategy, 12 cases of PMR following immunotherapy with nivolumab and pembrolizumab were included. Namely, 5 cases followed treatment with nivolumab and 7 with pembrolizumab. Nine patients were males.

In 2020, we published a systematic review that included both case series and case reports. Six reports involved patients in whom PMR was diagnosed following therapy with nivolumab or pembrolizumab. The lack of Naranjo’s scale or other validated algorithms for ADR assessment was identified as a significant critical flaw. Indeed, it was not clear whether ICIs triggered PMR, whether there was a genuine link between PMR and malignancies, or if both conditions merely coexisted [54]. This current systematic review reveals no clear ADR association; in about one-third of cases, this association was questionable, and no definite ADR was found [Table 1 and Table 2]. Despite recommendations from scientific societies [55], clinical judgment prevails to be the overriding criterion.

The 2018 Iskandar et al.’s case report deserves a comment. The authors reported on an 83-year-old man with metastatic melanoma who complained of PMR following the second cycle of treatment with pembrolizumab. Prednisone (20 mg orally daily, tapered over two weeks) was administered. The subject tolerated treatment with pembrolizumab well and did not require additional prednisone therapy. The authors calculated Naranjo’s total score of 8 for the association PMR-pembrolizumab [50]. However, when we recalculated this score using the same data as in the report, the result was 5: questions no. 4 and no. 5 had not been correctly scored. Additionally, their statement that “*treatment for PR typically consists of a short course of low dose steroids*” cannot be agreed by us in light of what is unanimously reported in the published literature [5,7]. In this respect, Iskandar et al.’s report strongly stresses the usefulness and opportunity of a closer collaboration between oncologists and rheumatologists.

The number of PMR following treatment with pembrolizumab was just over that of the number related to treatment with nivolumab (7 vs. 5). The time interval between PMR and nivolumab/pembrolizumab therapy ranged from one to 14 cycles. In almost all the case reports, the time of onset of PMR manifestations was fully compatible with the pharmacokinetics of nivolumab and pembrolizumab. Studies carried out on volunteers highlighted that: (a) serum clearance rate of pembrolizumab has a linear clearance in the first 77 days and a non-linear one after this period, which favors a faster removal of the drug from the human body [56]; (b) following infusion of nivolumab, PD-1 on circulating T lymphocytes is occupied by more than 70% (on average) for at least 2 months [57].

The case reported by Lobo et al. in 2020 was an exception worthy of being highlighted and discussed in detail. The authors diagnosed PMR in a patient with melanoma 11 months after completion of treatment with nivolumab [46]. However, when applying Naranjo’s scale to the published data of Lobo et al., the estimated total score was 0 (Table 1). It was, therefore, doubtful that PMR could be considered as an ADR in this patient, and the authors themselves would not exclude age-related, idiopathic PMR. To the best of our knowledge, PMR as delayed ADR following ICI therapy has never been reported again. Overall, delayed autoimmune toxicity occurring many months after cessation of anti-PD-1 therapy has very rarely been reported. Specifically, a case of autoimmune hepatitis was diagnosed almost 8 months after cessation of therapy with nivolumab in a 77-year-old female with metastatic melanoma [58]. The possibility of delayed autoimmune adverse events is thought to be linked to receptor occupancy by ICI that may be present even when ICIs are no longer dosable in the serum. This hypothesis is currently only speculative. Nivolumab and pembrolizumab pharmacokinetics authorized. Instead, the statement that PMR occurring after 3 months of completion of treatment with these two ICIs should not be considered as an ADR.

On the other hand, the observation that PMR can onset after a very short time interval from ICI administration, such as in the 2016 Garel et al. case report, suggests that the infusion might have only accelerated an already underway musculoskeletal inflammation. Specifically, these authors reported on an 88-year-old woman who complained of pain in the shoulders and hip girdles associated with morning stiffness > 1 h the day after the infusion of pembrolizumab 2 mg/kg [48]. The occurrence of a short time interval in a subset of ICI-PMR patients with mild inflammatory activity detected on 18F-FDG-PET/CT before ICI treatment has been reported in a recently published article [59].

To date, a shared and validated definition of PMR induced by ICIs (so-called “ICI-PMR”) is still lacking. Therefore, the question of whether ICI-PMR is a subset of disease or a PMR-like condition is still open [32,59,60]. Consequently, the distinction between true, idiopathic PMR and ICI-PMR is anything but easy in clinical practice. Referring to validated diagnostic or classification criteria for PMR [6,7,15,19] can significantly reduce the risk of misdiagnosis. In this regard, clinical judgment alone was the rule in all the case reports on pembrolizumab, with the only exception of Perrotta et al.’ s report [52]. If validated criteria for PMR were not used in nearly all case reports showing PMR in post-pembrolizumab treatment, how could the true PMR be differentiated from PMR-like conditions? Yet, the lack of an appropriate follow-up period (specifically, at least six months) was a further critical point emerging from our literature review. Indeed, in some patients initially diagnosed with PMR, diagnosis may change during follow-ups (precisely for the existence of PMR-like conditions). The consequences of misdiagnosis are easily understood: for example, a paraneoplastic syndrome may be mistaken for ICI-PMR (as in the report by Robilliard et al. [51] and in the report by Perrotta et al. [52]), or GC treatment could be started in cancer patients who did not need it. In conclusion, the possibility that PMR might be an umbrella term for every cancer patient with GC-responsive pain in the shoulder and pelvic girdle pain associated with long-lasting morning stiffness could not be excluded. A closer collaboration between oncologists and rheumatologists, referring to validated diagnostic or classification criteria for PMR, a shared definition of ICI-PMR, and a follow-up period of at least six months are often still unmet needs.

As of today, ICI-PMR pathogenesis is still speculative. Recently, we suggested as a working hypothesis that the first trigger was an antigenic stimulus (potentially activated by the primary or metastatic tumor mass) recognized and processed by the antigen-presenting macrophages. Subsequent activation of T-lymphocytes induced by ICIs could favor their infiltration in the anatomical sites where the inflammatory sequences of PMR are expected to start [60]. Based on our literature search, melanoma was present in 9 PMR patients, squamous NSCLCL in two patients and renal carcinoma in one patient. The role of cancer types, if any, in inducing an antigenic stimulus as the first step in the pathological cascade leading to PMR (as in the previously proposed hypothesis) was never investigated and has, therefore, to be clarified in further ad hoc research studies. According to our literature review, cancer status at the time-point of PMR diagnosis was not always the same: in most patients, PMR was diagnosed when cancer was in remission, or its status was unchanged; very rarely, PMR was diagnosed when cancer had metastasized. In short, there was no clear correlation between cancer status and PMR onset.

It is also interesting to highlight that the risk of PMR occurrence in cancer patients following treatment with nivolumab and pembrolizumab was not intra-class related. Indeed, when case reports of ICI-PMR following treatment with pembrolizumab were compared with those following treatment with nivolumab, differences were noted. In all case reports related to nivolumab, the drug was stopped, whereas pembrolizumab was not stopped in 2 patients. Additionally, validated PMR criteria were often used in the case reports related to nivolumab and almost never in the case reports related to pembrolizumab.

Lastly, a paraneoplastic PMR exacerbated by treatment with nivolumab has been reported [61]. Therefore, this possibility must always be excluded from clinical practice.

A final point deserves to be discussed. There are a number of scales and algorithms used to demonstrate a causal relationship between drugs and adverse clinical events, each with its own limitations and advantages. To date, Naranjo’s scale is the most widely used one, and it is easier to use in everyday clinical practice [62]. In addition, the use of this scale alone in our review was in line with both ISPE/ISoP guidelines and the EULAR expert panel [41,55]. Different assessment tools such as that proposed by WHO-UMC (the World Health Organization Collaborating Centre for International Drug Monitoring, the Uppsala Monitoring Centre) [63] are strongly based on the quality of the documentation of the observation, a quality that was unfortunately poor in the majority of the case reports included in this review.

Our review has strengths and limitations. To the best of our knowledge, this is the first systematic literature review of published case reports of PMR and PMR-like manifestations in cancer patients following treatment with nivolumab and pembrolizumab. Based on a recent systematic review, nivolumab and pembrolizumab are by far the most common ICIs involved in [32]. Inclusion and exclusion criteria were very selective and stringent, and this should be evaluated as a strength. Conversely, the small number of cases of PMR/PMR-like syndromes following treatments with nivolumab and pembrolizumab is a limitation, no doubt. Additionally, we were confined to what was documented in the included case reports. Specifically, the baseline of body mass index (BMI), comorbidities, and concomitant medications were almost always not reported. Therefore, the risk of potential biases in this documentation cannot be excluded.

## 5. Conclusions

Polymyalgia rheumatica has been reported in cancer patients following immunotherapy with nivolumab and pembrolizumab.

Our literature review identified several methodological blurred lines in the categorization of PMR and PMR-like syndromes following therapy with these ICIs. Specifically, the application of validated assessment tools (such as the Naranjo’s scale) was lacking in the majority of patients; validated diagnostic or classification criteria were lacking in almost all reports on PMR in post-pembrolizumab treatment; the follow-up duration was often too short or not reported; the possibility that ICI-PMR may be delayed ADR, occurring many months after cessation of nivolumab/pembrolizumab therapy, has yet to be convincingly proved.

Finally, a shared and validated definition of ICI-PMR is still lacking.

We hope that future reports will remove these grey areas and improve the quality of information.

## Figures and Tables

**Table 1:** The main characteristics of case reports of PMR following therapy with nivolumab.

1st Author, Year	Cancer	Onset of New, Isolated PMR	Atypical PMR Characteristics	PMR Diagnosis	PMR Criteria	Cancer Status	N. Stopped	Reintroduction	NS	NS Estimated Value	Follow-Up	Gender	Age
Bernier, 2017 [43]	squamous NSCL	4 cycles	MCP synovitis	Clinical + LF + US	not validated	Partial response	yes	no	n.a.	7	<3	M	70
Nakamagoe, 2017 [44]	melanoma	3 cycles	none	Clinical + LF + US + scint	2012 EULAR/ACR	Unchanged	yes	n.e.	n.a.	7	3 weeks	M	75
Imay, 2019 [45]	NSCLC	12 cycles	none	Clinical + LF + US + scint	2012 EULAR/ACR	Partial response	yes	n.e.	n.a.	7	n.r.	M	74
Lobo, 2020 [46]	melanoma	11 months after completion	none	Clinical + LF + CT scan	2012 EULAR/ACR	Regression	yes	n.e.	n.a.	7	>1 year	M	88
Moura, 2022 [47]	melanoma	14th cycle	none	Clinical + LF	not used	Regression	yes	n.e.	n.a.	7	>2 years	M	70

PMR = polymyalgia rheumatica; N = nivolumab; NSCLC = non-small cell lung cancer; n.e. = not evaluated; n.a. = not assessed; LF = laboratory findings; US = ultrasound; CT = computed tomography; M = male; NS Estimated Value = Naranjo’s scale Estimated Value.

**Table 2:** The main characteristics of the case reports of PMR following therapy with pembrolizumab.

1st Author, Year	Cancer	Onset of PMR	Atypical PMR Manifestations	PMR Diagnosis	PMR Criteria	Cancer Status	P Stopped	Reintroduction	NS	NS Estimated Value	Follow-Up	Gender	Age
Garel, 2016 [48]	melanoma	one cycle	none	Clinical + CT	not used	uncharged	Yes	not clear	No	5	>2 months	F	88
Garel, 2016 [48]	melanoma	3 cycles	n.c.	Clinical + Rx	not used	n.r.	No	n.r.	No	5	n.r.	M	79
Kuswanto, 2017 [49]	r.c.c.	4 cycles	knee arthralgias	Clinical + LF	not used	n.r.	Yes	n.r.	No	6	>1 year	F	68
Iskandar, 2018 [50]	melanoma	2 cycles	none	Clinical + LF	not used	uncharged	No	n.r.	Yes: 8	5	>2 weeks	M	83
Robilliard B, 2018 [51]	melanoma	4 cycles	none	Clinical	not used	metastasis	Yes	n.r.	No	6	not clear	F	79
Perrotta FM, 2020 [52]	melanoma	not clear	none	Clinical + LF + US	2012 EULAR/ACR	onset of breast cancer	Yes	No	Yes: 4	4	not clear	M	68
Kethireddy, 2020 [53]	melanoma	2 cycles	n.r.	Clinical + LF	not used	n.r.	Yes	No	No	5	7 years	M	85

PMR = polymyalgia rheumatica; CT = computed tomography; Rx = radiography; LF = laboratory findings; US = ultrasound; n.r. = not reported; r.c.c. = renal cell carcinoma; F = female; M = male; NS Estimated Value = Naranjo’s scale Estimated Value.

## Data Availability

No new data were created or analyzed in this study. Data sharing is not applicable to this article.

## Supplementary Materials

The following supporting information can be downloaded at: https://www.mdpi.com/article/10.3390/medsci13020034/s1, File S1: Full search strategy for OVID Medline database; File S2: Standardized form used to extract all required data.
