# Supplementary material for: Polymyalgia Rheumatica (PMR) and Polymyalgia Rheumatica-like (PMR-like) Manifestations in Cancer Patients Following Treatment with Nivolumab and Pembrolizumab: Methodological Blurred Points Identified Through a Systematic Review of Published Case Reports
Source: Med Sci (Basel). 2025 Apr 1;13(2):34. doi: 10.3390/medsci13020034 (PMC12015857; doi:10.3390/medsci13020034)
Supplement: Supplementary file 1 [file medsci-13-00034-s001.zip › medsci-3472970-File S1 Search strategy.pdf]

Database: Ovid MEDLINE(R) ALL <1946 to December 27, 2024>

Search Strategy:

1 Nivolumab/ (6064)

2 Nivolumab.af. (11533)

3 pembrolizumab.af. (11256)

4 polymyalgia rheumatica/ (2918)

5 ("rheumatic* polymyalgia" or "polymyalgia rheumatic*" or pmr).af. (7636)

6 exp Case Reports/ (2453600)

7 (1 or 2 or 3) and (4 or 5) and 6 (13)
