# Supplementary material for: Polymyalgia Rheumatica (PMR) and Polymyalgia Rheumatica-like (PMR-like) Manifestations in Cancer Patients Following Treatment with Nivolumab and Pembrolizumab: Methodological Blurred Points Identified Through a Systematic Review of Published Case Reports
Source: Med Sci (Basel). 2025 Apr 1;13(2):34. doi: 10.3390/medsci13020034 (PMC12015857; doi:10.3390/medsci13020034)
Supplement: Supplementary file 1 [file medsci-13-00034-s001.zip › medsci-3472970-File S2 Standardized ad hoc form.pdf]

**Table S1. General data**
First author
Title Journal
Year
Volume and Page numbers
Age
Sex

**Table S2. PMR criteria used**	
	Number
Chuang	
Bird	
Jones	
Nobunaga	
Healey	
Hunder	
2012 EULAR-ACR	
Not referenced	

**Table S3. Specific data**
Time interval between PMR and ICI:
Naranjo’s scale YES NO
Naranjo’s total score assessed using all data in the original report:
